# Onset and Progression of Human Osteoarthritis—Can Growth Factors, Inflammatory Cytokines, or Differential miRNA Expression Concomitantly Induce Proliferation, ECM Degradation, and Inflammation in Articular Cartilage?

**DOI:** 10.3390/ijms19082282

**Published:** 2018-08-03

**Authors:** Karen A. Boehme, Bernd Rolauffs

**Affiliations:** G.E.R.N. Tissue Replacement, Regeneration & Neogenesis, Department of Orthopedics and Trauma Surgery, Medical Center—Albert-Ludwigs-University of Freiburg, Faculty of Medicine, Albert-Ludwigs-University of Freiburg, 79085 Freiburg im Breisgau, Germany; karen.boehme@web.de

**Keywords:** early osteoarthritis, articular cartilage, proliferation, fibroblast growth factor 2, mitogen activated protein kinase, transforming growth factor β, SMA- and MAD-related protein, interleukin, nuclear factor kappa B, miRNA

## Abstract

Osteoarthritis (OA) is a degenerative whole joint disease, for which no preventative or therapeutic biological interventions are available. This is likely due to the fact that OA pathogenesis includes several signaling pathways, whose interactions remain unclear, especially at disease onset. Early OA is characterized by three key events: a rarely considered early phase of proliferation of cartilage-resident cells, in contrast to well-established increased synthesis, and degradation of extracellular matrix components and inflammation, associated with OA progression. We focused on the question, which of these key events are regulated by growth factors, inflammatory cytokines, and/or miRNA abundance. Collectively, we elucidated a specific sequence of the OA key events that are described best as a very early phase of proliferation of human articular cartilage (AC) cells and concomitant anabolic/catabolic effects that are accompanied by incipient pro-inflammatory effects. Many of the reviewed factors appeared able to induce one or two key events. Only one factor, fibroblast growth factor 2 (FGF2), is capable of concomitantly inducing all key events. Moreover, AC cell proliferation cannot be induced and, in fact, is suppressed by inflammatory signaling, suggesting that inflammatory signaling cannot be the sole inductor of all early OA key events, especially at disease onset.

## 1. Introduction

Osteoarthritis (OA) is a complex degenerative disease of the whole joint leading to progressive articular cartilage (AC) destruction. Even though multiple treatment guidelines have been proposed [1,2,3], no effective measures exist for the prevention of primary OA. True disease-modifying therapies for OA in the sense of a causal treatment are still missing. However, it is generally accepted that the level of damage occurring in early OA is potentially reversible [4] and that a better insight into the early OA mechanisms is likely the key for developing diagnostic strategies and targeted therapies [5,6]. 

**Figure 1:**
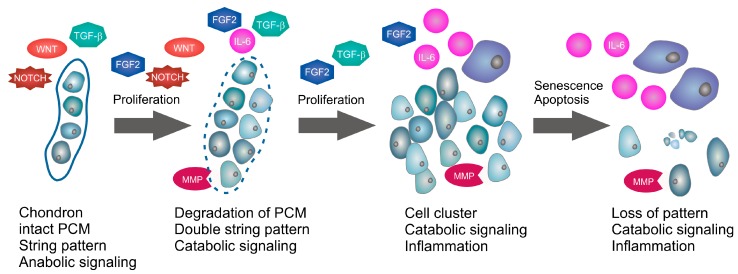
Human osteoarthritis onset and progression. Illustration of the relationship of signaling and superficial cell spatial organization (SCSO). Overview about key proteins in relation to changes in the SCSO of human articular cartilage (AC), which is based on the subsequent chapters that provide a detailed review of the individual pathways. In the superficial zone (SZ) of normal human adult AC differentiated chondrocytes are arranged in string patterns embedded in the pericellular matrix (PCM) and mediate extracellular matrix (ECM) maintenance. The onset of osteoarthritis (OA) is characterized by proliferation. During formation of double string patterns, the PCM is progressively degraded, presumably by MMPs and other catabolic factors. Proliferation in early OA is dependent on fibroblast growth factor 2 (FGF2), transforming growth factor β (TGF-β), wingless-type MMTV integration site family (WNT), and notch homolog (NOTCH) signaling, whereas catabolic matrix metalloproteinase (MMP) expression is mediated by FGF2, a switch in TGF-β signaling and inflammatory cytokines including IL-6. Subsequently, the processes maintaining sustained proliferation and ECM degradation lead to formation of cell clusters that develop from double strings. At the stage of SCSO clusters, pronounced inflammation outweighs attenuated growth factor impact. Late stage OA, accompanied by macroscopic ECM erosion, is characterized by senescence and apoptosis of cartilage-inherent cells and predominance of inflammation.

AC features a specialized architecture consisting of superficial (SZ), middle (MZ), and deep (DZ) zones [7,8], which are formed by modulation of the phenotype of the epiphyseal cartilage cells during skeletal growth and maturation [9]. Of particular interest, it has been shown that, when human AC samples are fluorescence-tagged and viewed from above, the cells exhibit complex patterns of arrangement in the surface layer of the superficial zone and with an orientation parallel to the joint surface, a feature that has been called superficial cell spatial organization (SCSO) [10,11]. The human SCSO can be highly dynamic, as horizontally orientated cell strings, for example in intact knee AC progress in early OA into double strings, together with an increased SZ cell density suggesting AC cell proliferation, are typical [12]. With OA progression, cell clusters occur, which are ultimately succeeded by a diffuse cell arrangement that is lacking any discernable organization [10,13]. A hallmark of early OA is proliferation of AC-inherent cells [10,13,14], which can be linked to these predictable SCSO changes (see Figure 1) through experimentally inducing AC cell proliferation in early OA AC explants beneath the joint surface. Indeed, proliferation induced via fibroblast growth factor 2 (FGF2) recapitulated SCSO loss and generated a structural AC phenotype that was comparable to advanced OA [13]: human AC explants containing strings and early OA-typical double strings oriented parallel to the surface altered their SCSO through induced proliferation into a diffuse arrangement lacking any discernable organization. Thus, early OA proliferation of SZ cells has a major impact on AC architecture. Moreover, AC cells that transiently proliferate during early OA and form clusters at the margins of extracellular matrix (ECM) fibrillation [14,15,16] express a large number of proteins that are involved in proliferation, ECM-degradation, and incipient inflammation [14,17], which illustrates the signaling complexity of early OA.

Indeed, research aimed at establishing a unified theory of the initial OA dysfunction so far has not been successful [18] and this is likely connected to the fact that OA pathogenesis includes several pathways, whose interactions remain unclear, especially at the onset of the disease [19]. The current review focused on the relationship between the signaling pathways that are associated with rarely considered proliferation of human AC cells in early OA and the anabolic, catabolic and pro-inflammatory effects that are well-established and have been associated with OA progression. More specifically, the review focused on the questions, which of the three key events in AC—proliferation, ECM degradation, and inflammation—are inducible by growth factor signaling, inflammatory cytokine signaling, and/or miRNA regulation. Additionally, we aimed to reveal in which sequence(s) these events can and cannot occur, and whether we can identify a single factor that is able to induce all of these key events, according to the currently available knowledge.

The two most examined pro-inflammatory cytokines in early OA are Interleukin-1 beta (IL-1β) and tumor necrosis factor α (TNF-α) [20] but various other pro-inflammatory cytokines and chemokines such as IL-6, IL-8, and IL-17 may also be involved in early OA pathology [20,21]. Proliferation of AC cells is modulated by fibroblast growth factor 2 (FGF2) [22,23] and transforming growth factor β (TGF-β) [24] signaling, in addition to many other effects. Therefore, particularly FGF2, TGF-β, and inflammatory cytokine signaling in combination with their miRNA regulation in human AC have been reviewed.

## 2. Fibroblast Growth Factor 2 Signaling

**Figure 2:**
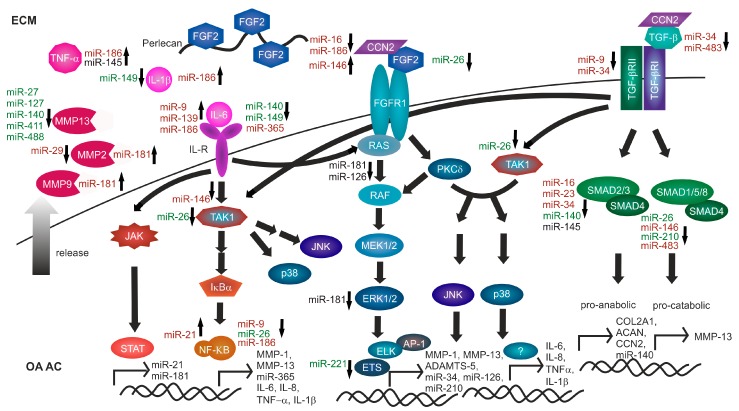
FGF2, TGF-β and inflammatory cytokine induced catabolic and pro-inflammatory signaling in human OA AC. The major components of inflammatory cytokine, FGF2 and TGF-β activated nuclear factor kappa B (NF-κB), mitogen activated protein kinase (MAPK), and SMA- and MAD-related protein (SMAD) signaling as well as their transcriptional targets in human OA AC are depicted. Micro RNAs (miRNAs) upregulated in human OA AC are indicated in red, miRNAs downregulated in human OA AC are indicated in green. For miRNAs with contradictory regulation a black font is chosen. The small arrows beside the miRNAs pointing downwards indicate direct or indirect inhibition of the signaling component by the miRNA, whereas small upward arrows indicate direct or indirect activation of the signaling component by the miRNA. As illustrated, there is an intense crosstalk between the individual pathways. In human OA AC, expression of pro-catabolic target genes is mediated by NF-κB, MAPK and SMAD signaling, whereas pro-inflammatory targets are activated downstream of MAPK and NF-κB signaling. Advanced stages of OA are characterized by activation of inflammatory cytokine signaling. On miRNA level there is primarily a net upregulation of IL-6, whereas both positive and negative regulation of NF-κB signaling is reported. Moreover, in human OA AC upregulation of CCN2 and significantly enhanced activation of extracellular signal-regulated kinase (ERK), JUN N-terminal kinase (JNK) and p38 MAPK pathways has been documented. Yet, evidence for ERK signaling regulation by miRNA is contradictory, whereas JNK and p38 activation is a common downstream event after inflammatory cytokine, FGF2 and TGFβ pathway activation with no direct miRNA regulation reported in OA AC to date. TGF-β signaling is globally downregulated in OA AC with especially negative regulation of the anabolic SMAD3 mediated pathway which is both evident on protein and miRNA level. In addition, in particular at miRNA level, reversal of negative regulation of MMP-13 is obvious in OA AC.

FGF2 is participating in several signaling pathways regulating proliferation, migration, inflammation, angiogenesis, differentiation, and senescence [22,23]. FGF2 is produced endogenously in human AC and occurs bound to perlecan, a heparan sulfate proteoglycan (HSPG) in the pericellular matrix (PCM) [25] (see Figure 2). Upon cutting of human AC, FGF2 is released from the PCM, activating mitogen activated protein kinase (MAPK) signaling [26]. Interestingly, FGF2 induces proliferation in both human intact and OA AC [27]. Moreover, FGF2 transduction of human knee AC samples is capable of recapitulating SCSO changes observed in early OA by inducing AC cell proliferation, which cumulates in complete SCSO loss comparable to an advanced OA-like structural phenotype of human AC [13]. In addition, FGF2 expression has been described as a marker of the human AC mesenchymal stem and progenitor cell (MSPC) population and is implicated in MSPC proliferation and chondrogenesis [23]. Besides, FGF2 acts as a chemo-attractant for monocytes and can be released by a variety of immune cells [28]. The FGF2 concentration in plasma and knee synovial fluid (SF) of OA patients is approximately twice of that of patients with normal healthy knee joints. Moreover, the increase of FGF2 abundance in OA plasma and SF correlates positively with radiographic OA severity [29]. Cells in human healthy and OA AC express all four fibroblast growth factor receptors (FGFR), but FGFR1 and FGFR3 dominate by far, compared to FGFR2 and FGFR4 [30,31]. Moreover, in human OA AC cells FGFR1 expression is increased while FGFR3 is concomitantly suppressed, compared to healthy AC cells [30].

In monolayer cell cultures established from human healthy AC, rFGF2 stimulation independently activates both protein kinase C δ (PKC δ) and rat sarcoma viral oncogene homolog (RAS) signaling cascades [32] predominantly via FGFR1 [33]. In parallel, the extracellular signal-regulated kinase (ERK), p38, and JUN N-terminal kinase (JNK) MAPK pathways are activated by PKC δ [32], whereas RAS predominantly activates the ERK signaling cascade [34] (see Figure 2). ERK phosphorylation is enhanced in human OA AC compared to healthy AC [35,36]. Moreover, human OA AC shows higher phosphorylated and therefore activated p38 MAPK level compared with normal AC [36,37]. Also, JNK activation is enhanced in human OA AC, compared to healthy control AC [36]. Notably, the highest phosphorylation of all MAPKs is found in the SZ of both healthy and OA AC [38]. 

The MAPK signaling cascade appears to be the dominating pathway responsible for matrix metalloproteinase (MMP)-1 and MMP-13 mRNA and protein expression in human healthy and OA AC cells in response to rFGF2 [32,39]. MMP-13 transcription in human (OA) AC cells in response to rFGF2 can be mediated by the transcription factor ETS-domain protein ELK-1 (ELK1) [32,35]. rFGF-2 dependent upregulation and activation of MMP-9 regulated by RAS and PKC δ dependent MAPK activation has been reported utilizing a human breast cancer cell line, whereas MMP-2 is not affected by rFGF2 in these cells [40]. In addition, in rat costal chondrocytes, rFGF2 dependent activation of MMP-9 has been reported [41]. Yet, in human AC, the impact of FGF2 induced MAPK signaling on MMP-2 and MMP-9 expression and activation has not been elucidated so far.

In cells isolated from human macroscopically healthy AC and OA AC, rFGF2 also suppresses expression of aggrecan (ACAN) and collagen type II α 1 chain (COL2A1) [30,39]. Important in the context of this review, rFGF2 promotes the expression of the inflammatory cytokines *TNF-α*, *IL-1β*, *IL-6*, *IL-8*, and *monocyte chemotactic protein 1* (*MCP-1*), also known as *C-C motif chemokine ligand 2* (*CCL2*) [32], highlighting an important pro-inflammatory role of FGF2. Moreover, rFGF2 upregulates runt related transcription factor 2 (RUNX2) activity in bovine, murine and human cells of different mesenchymal tissue origin, which, in turn, controls *collagen type X α 1 chain* (*COL10A1*), *MMP-13* and *A disintegrin and metalloproteinase with thrombospondin motifs* (*ADAMTS*)*-5* at the level of transcription [42,43,44,45]. Interestingly, treatment of human AC cells from young and healthy donors (Collins grade 0 or 1, <35-year-old) with rFGF2 shows no significant anti-anabolic or catabolic effect; rFGF2 fails to repress ACAN expression or induce MMP-13 and ADAMTS-5 expression in these cells. By contrast, notable effects on expression of these genes are observed when the same dose of rFGF2 is applied to damaged AC from older donors (grade 2 or higher, >40-year-old) [33]. These findings suggest a contextual property of FGF2 in AC biology, probably mediated by changes in abundance and activity of FGFR and other downstream components of FGF2 signaling. Constitutive rFGF2 expression after recombinant *adeno-associated virus* (rAAV)-hFGF2 transduction of human early OA AC explants induces cell proliferation within the native tissue [13]. Also, in monolayer cultures of human OA AC cells, rFGF2 enhances proliferation and prevents cell death [46].

In contrast to the above discussed human signaling profile showing predominant expression of FGFR1 and FGFR3, in murine healthy and surgically induced OA AC Fgfr2 and Fgfr4 are predominantly expressed, while Fgfr3 is barely detectable [31]. Surgical induction of OA in murine AC slightly reduces the expression of all Fgfr subtypes, but rFgf2 local injection markedly induces Fgfr3 expression, which is opposite to the human OA scenario [30,31], where rFGF2 selectively reduces FGFR3 expression. Indeed, Fgf2 has anabolic functions in murine AC that are mediated by Fgfr3. This is in strong contrast to the rFGF2-mediated anti-anabolic and catabolic in human aged healthy and OA AC [34]. In murine OA models rFgf2 mediates proteoglycan deposition in AC [31,47]. In addition to its species-dependent effects, the AC protective activity of rFGF2 in animal models appears to be age-dependent, too, as seen in rabbit [48] and bovine AC [49], where the anabolic activity is restricted to AC from young animals. Moreover, in calf AC only low doses of 3 ng/mL FGF2 induce proliferation, whereas higher doses of 30–300 ng have no mitotic effect [49]. FGF2 adaptor proteins like CCN2, also known as connective tissue growth factor (CTGF), may fine tune FGF2 signaling in mammalian AC [41]. CCN2 mRNA and protein overexpression has been shown in human OA AC compared to healthy AC [50,51]. 

Thus, FGF-2 mediates proliferation, anti-anabolism, and catabolism in human AC. However, healthy cells of young donors appear to be resistant against the catabolic effects of FGF2. The important ability of FGF2 to induce inflammatory cytokine expression in human AC cells isolated from macroscopically healthy, but aged AC may be sufficient to induce or reinforce inflammation, dependent on the context and, thus, trigger OA progression.

## 3. Transforming Growth Factor β Signaling

TGF-β family ligands are growth factors basically implicated in proliferation, differentiation, and ECM maintenance. Binding to their hetero-tetrameric receptor, consisting of type I and type II subunits (TGF-βR1, TGF-βR2), activates TGF-β signaling [24]. Expression of the three TGF-β isoforms and both receptor subtypes has been examined in human OA AC compared to macroscopically healthy AC. However, the results are contradictory. While an upregulation of TGF-β1, TGF-β3, and TGF-β-R2 proteins with increased severity of OA has been reported in hip AC [52,53], downregulation of TGF-β1 protein in knee OA AC has been observed [54]. In addition, a polymorphism in the *asporin* (*ASPN*) gene, leading to reinforced TGF-β1 inhibition, has been associated with increased OA susceptibility [55,56]. Also, a single nucleotide polymorphism (SNP) in the *SMA-* and *MAD-related protein 3 (SMAD3)* gene has been linked with an increased risk of hip and knee OA [57].

In healthy adult AC cells all TGF-β isoforms induce proliferation, with an age dependent decline in responsiveness [58]. Moreover, anabolic expression of *COL2A1* and *ACAN* has been reported in response to rTGF-β1 and rTGF-β2 in human healthy AC cells [59] (see Figure 2). 

Studies with human OA AC cells show that in OA TGF-β signals predominantly through activin receptor-like kinase 1 (ALK1)/activin A receptor like type 1 (ACVRL1) SMAD1/5/8 pathways, which is linked to the induction of catabolism; e.g., *MMP-13* expression [60,61]. Indeed, it is commonly suggested that ageing or onset of OA switch the receptor in TGF-β signaling from the classical ALK5/TGF-β-R1 activated Smad2/3 signaling to TGF-β-R1 family member ALK1/ACVRL1 induced SMAD1/5/8 signaling, which converts TGF-β function in AC from an anabolic growth factor into a catabolic cytokine [62]. 

However, in OA AC both ALK1 and ALK5 expression appears to be largely reduced compared to healthy AC, although with a relative ALK1 excess [63]. rAAV-mediated TGF-β expression induces proliferation and proteoglycan deposition in both human healthy and OA AC cells, while increasing both ALK1 and ALK5 expression, leading to an elevated, balanced receptor expression in OA AC cells resembling healthy AC. Indeed, MMP-13 protein expression is largely reduced in OA AC cells by this approach, while COL2A1 expression increases, indicating simultaneous anabolic and anti-catabolic actions of prolonged TGF-β expression in human OA AC [63].

Although SMAD signaling is dominating in the TGF-β response, additional downstream signaling includes activation of TGF-β-activated kinase-1 (TAK1), also known as mitogen-activated protein kinase kinase kinase 7 (MAP3K7), which acts as upstream activators of MAPK signaling and nuclear factor kappa B (NF-κB) signaling in OA AC, though its role in human AC is not well-investigated [64,65]. Moreover, CCN2 is a transcriptional target of TGF-β and MAPK signaling. Interestingly, in human fibroblast cultures CCN2 is necessary for the TGF-β induced phosphorylation of SMAD1 and ERK1/2, but it is dispensable for activation of the SMAD3 pathway [66]. Yet, in human AC implication of these pathways in CCN2 expression has not been validated so far [67]. 

Summarized, in human healthy AC TGF-β signaling induces proliferation and anabolic gene expression via ALK5, a function which declines with increasing age. In contrast, in OA AC a pathway switch to ALK1 receptor converts TGF-β from an anabolic cytokine into a catabolic factor promoting AC degradation. In the context of inflammation, TAK1 activation, a downstream event of several signaling pathways including FGF2 or TGF-β signaling (Figure 2) has the potential to induce pro-inflammatory gene expression. Indeed, in a rat OA model, intra-articular injection of Tak1 adenovirus induced the secretion of several pro-inflammatory interleukins in the synovial fluid [68]. However, until today, no pro-inflammatory function of TGF-β signaling has been determined in human AC.

## 4. Additional Growth Factor Signaling Pathways in Human Adult AC

In addition to FGF2 and TGF-β signaling, several other growth factors and their downstream pathway components appear to be expressed in human adult AC. The differential regulation of these growth factor-induced signaling pathways as well as their impact on proliferation, anabolic/catabolic gene expression, or inflammation in human OA AC is summarized in this chapter.

### 4.1. WNT Signaling

Evidence for progressive activation of the non-canonical Ca^2+^/wingless-type MMTV integration site family (WNT) signaling pathway in human OA AC is provided by increased expression of WNT5A mRNA and protein [69,70,71] as well as *CaMK2 nuclear factor of activated T-cells 5*, *tonicity-responsive (NFAT5)*, and *nuclear factor of activated T-cells 2 (NFATC2)* mRNA [70]. WNT5A protein expression in healthy human AC is restricted to the SZ, whereas in OA AC also cells of the deeper layers express WNT5A. Indeed, in human normal AC monolayer cultures rWNT5A promotes repression of anabolic genes like *ACAN*, whereas mRNA expression of catabolic genes including *MMP-1*, *MMP-3*, *MMP-13* and *ADAMTS-4* is enhanced [69,72]. In addition, rWNT5A induces MMP-1 and MMP-13 protein expression [69].

While *WNT7A* mRNA expression is downregulated in human OA AC compared to normal AC, ectopic lentiviral expression of rWNT7A in human normal AC cell cultures inhibits rIL-1β-induced catabolic gene expression including *MMP-1*, *MMP-3* and *MMP-13*, which is likely mediated via the non-canonical Ca^2+^/WNT signaling pathway [73]. 

Also, *WNT3A* mRNA expression is increasingly downregulated with higher grades of OA severity in human AC [74]. rWNT3A promotes dedifferentiation, indicated by loss of anabolic *COL2A1* and *ACAN* expression in human OA AC monolayer cultures, which is mediated by the Ca^2+^/calmodulin-dependent protein kinase 2 (CaMK2) pathway [74]. Indeed, rWNT3A-dependent induction of proliferation, but also *axis inhibition protein 2 (AXIN2)* expression, an inhibitor of canonical WNT signaling, is specifically mediated by the canonical WNT/β-catenin pathway in human OA AC cells [74]. Moreover, in another study using rWNT3A in human OA AC cell monolayer cultures, activation of canonical WNT/β-catenin signaling has turned out to be a potent inhibitor of *MMP-1*, *MMP-3*, and *MMP-13* expression and MMP activity both under basal conditions and also after rIL-1β stimulated NF-κB activation [75]. This indicates the ability of WNT3A to induce proliferation in human adult AC, whereas catabolic gene expression is actively repressed.

Interestingly, in human OA AC mRNA and protein expression of intracellular and extracellular inhibitors of both the canonical and planar cell polarity WNT pathways (e.g., AXIN2), as well as dickkopf WNT signaling pathway inhibitor 1 and 3 (DKK1 and DKK3), are significantly upregulated compared to normal AC [70,76]. AXIN2, DKK1, and DKK3 proteins in normal human AC are predominantly localized to the SZ, whereas their expression is extended to the cells located in the deeper layers of human OA AC [70]. Yet, another study shows reduced *DKK1* mRNA expression in human OA AC with increased OA grading [77]. However, despite obviously enhanced expression of canonical WNT signaling inhibitors upon onset of OA [70], increased nuclear localization of β-catenin protein occurs in human early and late OA AC, compared to normal control AC, indicating sustained activation of canonical WNT signaling in OA AC [78].

Human female hip and knee OA has been associated with a polymorphism in the *frizzled related protein (FRZB)* gene [79,80,81]. FRZB functions as a soluble WNT decoy receptor and, thus, can inhibit canonical and non-canonical WNT signaling pathways [82]. Interestingly, a FRZB double mutant associated with human AC OA exhibits decreased affinity for WNT molecules, suggesting a compromised ability to suppress WNT signaling [82]. In addition, decreased *FRZB* mRNA expression in human AC has been associated with increased OA grading [77].

Summarized, WNT signaling in human adult AC is complex. Whereas canonical WNT/β-catenin signaling may play a proliferation-inducing and anti-catabolic role in human healthy and early OA AC, the Ca^2+^/CaMK2 arm of WNT signaling may induce dedifferentiation and catabolic gene expression. Progression of OA apparently depends on the balance of inhibition and activation of different WNT pathways, ultimately leading to cessation of proliferation and increased catabolism. To date, there is no evidence for a pro-inflammatory activity of WNT signaling in human AC.

### 4.2. Hedgehog Signaling

Indian hedgehog (IHH)-induced hedgehog (Hh) signaling is a key pathway implicated in proliferation and differentiation during vertebrate AC development and longitudinal growth at the growth plate [83,84,85]. In human OA AC and OA SF, IHH abundance is increased compared to normal controls. Indeed, IHH protein expression is already enhanced in early human OA AC lesions and its expression increases with OA severity. In contrast, in the SF of late stage OA patients, the IHH protein content declines, compared to early OA [86,87]. Interestingly, the IHH protein is predominantly located in the SZ of human OA AC [86]. Additionally, in human knees categorized with the most severe OA, AC expresses the highest levels of Hh downstream target genes *glioma-associated oncogene homolog 1 (GLI1)*, *patched 1* (*PTCH1)* and *hedgehog interacting protein* (*HHIP)* [88]. rIHH activates Hh signaling in human first passage monolayer cultures derived from normal adult AC without inducing catabolic *ADAMTS-5* or *MMP-13* expression. These results show that IHH signaling by itself does not cause catabolic ECM degradation in human normal AC [89]. Indeed, the lack of catabolic response to IHH signaling in healthy AC is in contrast to another study demonstrating in human OA AC explants that recombinant sonic hedgehog (SHH), another Hh ligand, induces catabolic *ADAMTS-5* mRNA expression [88]. 

In short, the outcome of Hh signaling activation during vertebrate AC development and longitudinal bone growth has been determined in many studies. However, the impact of apparent Hh pathway component overexpression on human adult AC proliferation remains to be elucidated. In addition, the mechanistic background of Hh signaling-induced catabolic gene expression in human OA AC has to be resolved by additional research. Until today, there is no evidence for any pro-inflammatory activity of Hh signaling in human AC.

### 4.3. Bone Morphogenetic Protein Signaling 

Bone morphogenetic protein (BMP) signaling, like Hh signaling, has a central function in vertebrate cartilage development, stimulating both proliferation and anabolic gene expression [90]. Also, in human adult AC BMP ligand expression can be detected. BMP-2 mRNA and protein expression is up-regulated in OA AC and OA AC derived monolayer cell cultures [91,92,93,94]. Indeed, *BMP-2* mRNA expression can be detected in OA AC cell clusters and single cells of the SZ and MZ. Only in severely damaged AC, *BMP-2* mRNA is also located in the DZ [92]. In primary cultures of human OA AC cells BMP-4 is upregulated compared to normal control AC cells [93]. Also, BMP-1, BMP6, and BMP-11 expression is abundant in human adult AC, but without apparent differential regulation upon OA onset [95,96]. In contrast, BMP-3 and BMP-7 are clearly downregulated in human OA AC, although data concerning BMP-7 expression are contradictory. One study found both BMP-3 and BMP-7 predominantly expressed in the SZ of normal AC. Moreover, BMP-7 was detected in early OA AC cell clusters, whereas BMP-3 expression was absent upon OA onset [97]. In another study, both human normal and OA AC lacked BMP-7 protein expression, which was only detected in fetal, developing AC [96]. rBMP-2 induces anabolic gene expression, including *ACAN* and *COL2A1* as well as increased proteoglycan deposition in human normal adult AC (both young and aged) cell cultures as well as OA AC cell cultures [91,98,99,100,101]. Expression of catabolic *MMP-2* and *MMP-3* mRNA is not affected by rBMP-2 [99]. Yet, another study in human OA AC monolayer cultures shows rBMP-2 induced catabolic *MMP-9*, *MMP-13* and *ADAMTS-5* mRNA expression, which is at least partially mediated by WNT/β-catenin signaling [93]. Interestingly, the proteoglycan synthesis induced by rBMP-6 in normal adult AC derived monolayer cultures shows an age dependent decrease. Also, OA AC cell cultures exhibit a limited proteoglycan deposition upon rBMP-6 comparable to aged normal AC [95]. rBMP-7 specifically promotes anabolic *ACAN* and *COL2A1* mRNA expression in human adult OA AC cell high density monolayer cultures. Yet, expression of catabolic *MMP-1*, *MMP-3*, *MMP-13* and *ADAMTS-4* genes is not affected by rBMP-7 [102] or in case of human adult AC cell alginate bead cultures even reduced [100]. No positive effect of rBMP-2, rBMP-4, rBMP-6 or rBMP-7 on proliferation of human adult AC cell monolayer or alginate bead cultures was observed [95,100]. In addition, there is no indication that BMP signaling can promote inflammation in human OA AC, whereas rIL-1β and rTNF-α increase BMP-2 mRNA and protein levels in human OA AC explant cultures [91]. Yet, in the context of rheumatoid arthritis, BMP signaling may have anti-inflammatory functions [103]. 

Summarized, in human adult normal and OA AC, the outcome of BMP signaling is anabolic and potentially also catabolic, via a cross-talk with canonical WNT signaling. However, there is no evidence for a pro-proliferative or inflammation-inducing function.

### 4.4. NOTCH Signaling

In human macroscopically intact adult AC, notch homolog (NOTCH) receptors and ligands are scarcely expressed. However, in human OA AC mRNA and protein expression of all four NOTCH receptors, jagged 1 (JAG1) and delta-like 1 (DLL1) ligands as well as hairy and enhancer of split 1 (HES1) and HES5 are abundant, especially in cell clusters within the SZ [104,105,106,107]. Moreover, proliferation of human OA AC cell cultures in vitro is induced by and depends on active NOTCH signaling [105]. In monolayer cultures of human OA AC cells, NOTCH signaling represses the expression of *BMP-2*, which is implicated in anabolic gene expression. Simultaneously, the expression of pro-inflammatory and catabolic genes, including *IL-8* and *MMP-9*, is repressed by active NOTCH signaling [105]. 

Taken together, NOTCH signaling appears to be activated specifically in human OA AC and to contribute to increased proliferation, whereas it likely inhibits catabolic and inflammatory gene expression. 

### 4.5. Insulin-Like Growth Factor Signaling

In normal human adult AC insulin like growth factor 1 (IGF-1) is predominantly localized in the SZ. Intriguingly, both in human OA AC and OA SF the IGF-1 protein concentration significantly increases [108,109]. Both in monolayer cultures and explants of human normal adult AC rIGF-1 has pro-proliferative and anabolic effects, indicated by increased proteoglycan synthesis and expression of collagen type II [110,111]. Interestingly, rFGF2 dose dependently antagonizes rIGF-1-mediated proteoglycan deposition in human normal AC alginate cultures, whereas both promote proliferation [112]. For human OA AC no data concerning IGF-1 signaling outcome are available. 

Summarized, in human normal adult AC, IGF-1 has mitogenic and anabolic functions. Until today, IGF-1 signaling has neither been implicated in human AC catabolic gene expression nor in inflammation.

### 4.6. Vascular Endothelial Growth Factor Signaling

Angiogenesis mediated by vascular endothelial growth factor (VEGF) is a contributing factor in OA pathogenesis. Yet, angiogenesis, comprising catabolic ECM degradation and endothelial cell proliferation, remains restricted to tissues such as the synovium and the subchondral bone, whereas AC itself remains avascular during OA progression [113]. Nevertheless, VEGF A is actively expressed in human adult AC. In human normal and OA AC the mRNAs of three VEGF A isoforms (*VEGF121*, *VEGF165*, and *VEGF189*) can be detected and VEGF protein is predominantly localized in the SZ and MZ of OA AC, both intracellularly and in the PCM [114,115,116]. Intriguingly, an upregulation of VEGF expression in OA AC compared to normal adult AC has been reported [116,117,118]. Expression of the VEGF receptors VEGFR-1, also known as Fms related tyrosine kinase 1 (FLT-1) and VEGFR-2, also known as kinase insert domain receptor (KDR) is either restricted to OA AC compared to normal AC [115], whereas other studies reported that VEGFR-1 [116] or VEGFR-2 [119] were not detectable at all in human adult AC. Moreover, in human OA AC VEGFR-3, also known as Fms-like tyrosine kinase 4 (FLT4), is expressed in the SZ cells located in cytoplasm and on cell membrane [120]. In primary OA AC monolayer cultures catabolic *MMP-1* and *MMP-3* mRNA expression, but not *MMP-2*, *MMP-9* or *MMP-13* expression, can be induced by rVEGF165, whereas cultured normal AC cells exhibit no catabolic gene expression upon rVEGF165 treatment at all [115]. No proliferation-inducing effect can be attributed to rVEGF in human OA AC cells [115,119]. 

In summary, active VEGF signaling appears to be restricted to human OA AC, where its outcome is catabolic. Yet, proliferation of human OA AC cells is not affected by VEGF. Until today, no VEGF induced expression of pro-inflammatory genes in human OA AC has been reported. Nevertheless, in other tissues pro-inflammatory VEGF action is renowned [121]. 

## 5. Inflammatory Cytokine Signaling

It is well-accepted that inflammation is ubiquitous during OA progression [122]. Yet, it is being debated whether inflammation may also be a primary conductive trigger for the onset of OA [123,124].

IL-1β and TNF-α are the best-studied pro-inflammatory cytokines in human AC experimental OA induction [21]. Apart from that, also IL-6, the IL-6 like cytokine oncostatin M (OSM), IL-17, and IL-8 have been implicated in human OA pathogenesis [20,125]. Yet, IL-1β, which has been discovered as AC destructive factor in porcine tissue, is apparently not substantially upregulated in the human SF of joints with different OA stages [125,126]. Moreover, in SF of OA patients the abundance of IL-1 receptor antagonist (IL-1Ra), competing with IL-1β for IL-1 receptor (IL-1R) binding, was 1800 times higher compared to IL-1β [125], whereas the IL-1R density on human OA AC cells was less than a 2-fold increased [127]. Together, these findings suggest in human OA SF in vivo an inhibition of IL-1β signaling at endogenous IL-1β concentrations [125]. 

Increased expression of IL-17a, IL-8, monokine induced by interferon-gamma (MIG) and interferon-inducible T-cell alpha chemoattractant (I-TAC) has been found in SF and serum of OA patients [128]. Another study identifies IL-6, but also IL-1β and TNF-α protein, to be specifically upregulated in serum of OA patients [36]. Moreover, *IL-6* mRNA expression is enhanced in human fibrillated OA AC, whereas no *IL-6* signal was evident in histologically normal AC from OA patients or healthy human control AC [129]. Interestingly, this study also revealed that IL-1β expression in human AC did not to correlate with the presence or grading of OA, whereas another study reported decreased IL-1β protein abundance in human OA AC with increased OA grading [130].

rIL-1β stimulates the expression of MMP-1, MMP-3, and MMP-13 mRNA and protein in human healthy and OA AC monolayer cultures [42,131]. In addition, rIL-1 induces IL-8 release from human OA AC cells [132]. Also, *IL-6* and *FGF2* mRNA expression are markedly enhanced by rIL-1β in human healthy AC cell cultures [131]. Indeed, rIL-17 is able to induce IL-8, IL-1β and IL-6 protein release in human healthy and OA AC cells [132,133]. In addition, rIL-8 upregulates secretion of IL-1β, IL-6, TNF-α, MMP-1, MMP-3, and MMP-13 by human OA AC cells [128]. Interestingly, in human OA AC cell cultures depletion of IL-6 prevents rIL-1β-induced MMP-13 expression [134], underlining the possibility that IL-1β may not be the primary cytokine involved in OA AC inflammation. Although human healthy and OA SF contains about 20 pg/mL IL-1β and less than 3 ng/mL TNF-α, many studies using inflammatory cytokines for experimental OA induction apply apparently supra-physiological doses of 1–100 ng/mL rIL-1β and up to 50 ng/mL rTNF-α for activation of downstream signaling [21,125], which may probably not reflect the natural OA pathogenesis in vivo [21]. 

In human AC, the signaling cascades originating from IL-1β and TNF-α converge on MAPK and NF-κB signaling [135] (see Figure 2). NF-κB in cooperation with ERK, p38 and JNK regulate rIL-1β and rTNF-α-dependent catabolic *MMP-13* production in human OA AC cells via E74-like factor 3 (ELF3) and activator protein 1 (AP-1) [136,137,138]. Knockdown of NF-κB p65/RelA suppresses the expression of basal and rIL-1β-induced *MMP-1* and *MMP-13* mRNA in human OA AC cells [75]. 

Notably, IL-17 activates ERK, JNK and p38 as well as NF-κB in normal human AC cells to induce *IL-1β* and *IL-6* expression [133]. Intriguingly, in human OA AC cells, rIL-17 activates FBJ murine osteosarcoma viral oncogene homolog B (FOSB) (AP-1 subunit), whereas IL-1β activates cellular oncogene Fos (cFOS) (AP-1 subunit) to induce MMP-13 release, indicating a different fine tuning of downstream signaling depending on the cytokine [139]. In addition, rIL-8 activates NF-κB and JNK signaling in human OA AC cells to induce IL-1β, IL-6, TNF-α, MMP-1, MMP-3, and MMP-13 secretion [128]. 

Overall, there is an intense crosstalk of inflammatory cytokine signaling with different growth factor-induced signaling pathways. 

Interestingly, in human OA AC, rIL-1β treatment simultaneously down-regulates mRNA expression of the WNT signaling inhibitors *FRZB* and *DKK1*, whereas *WNT5A* mRNA expression is increased by rIL-1β treatment both in human healthy and OA AC. While *FRZB* downregulation and *WNT5A* overexpression have also been observed in OA patients, data for DKK1 expression in OA patients are contentious [70,77]. In human OA AC cells canonical WNT/β-catenin signaling, activated by rWNT3A, counteracts NF-κB-mediated *MMP* expression induced by rIL-1β. Additionally, rWNT3A inhibits rIL-1β induced *IL-6* expression [75], indicating an attenuating role of canonical WNT signaling on human AC inflammation.

Both rTNF-α and rIL-1β significantly repress mRNA expression of several NOTCH pathway components, including *NOTCH1*, *NOTCH3*, *JAG1*, and *HES5* in human healthy and OA AC cells in vitro [105]. Intriguingly, these proteins have been shown to be upregulated in human OA AC in vivo [104,105], revealing the absence of inflammatory cytokine-mediated suppression of NOTCH signaling at least during early OA in vivo. 

Hh pathway activation is suppressed by addition of rIL-1β in adult bovine AC explants. Conversely, rIHH weakly suppresses rIL-1β-induced *ADAMTS-5* expression in this model [89], indicating a negative feedback of both pathways. In healthy human AC cell alginate bead cultures rIGF-1 upregulates IL-1RII protein expression, a decoy receptor for IL-1, which may override catabolic IL-1β actions in healthy AC [140]. 

Remarkably, FGF2 is the only growth factor considered, which directly promotes the mRNA expression of *TNF-α*, *IL-1β*, *IL-6*, *IL-8,* and *MCP-1* in human healthy AC cells, thereby promoting inflammation after 5 days of monolayer culture [32]. On the other hand, in healthy human AC and in the murine teratocarcinoma ATDC5 cell line, rIL-1β increases FGF2 mRNA and protein expression [32,141], indicating a potential feedback loop between FGF2 and Il-1ß.

As discussed, proliferation-induced changes in the SCSO of human AC and formation of cell clusters are early OA marker [10,14]. Increased expression of the proliferation markers *cyclin D1* (*CCDN1*) and *cyclin dependent kinase 6 (CDK6)* has been observed in human OA AC compared to healthy control AC [142]. In addition to FGF2, growth factors like IGF-1, NOTCH ligands, and WNT have also been implicated in human adult AC cell proliferation [13,74,105,112]. In contrast, inflammatory cytokines clearly inhibit adult human AC cell proliferation. This is obvious in human OA AC for rIL-1β, which inhibits proliferation and induces apoptosis [36], but also for rIL-8, which even suppresses proliferation [128]. Moreover, in rabbit AC rIL-6 represses proliferation [143]. Interestingly, in healthy human AC cell agarose bead cultures rIL-1β also represses rFGF2-induced proliferation and cluster formation; only IL-17R overexpression has been associated with increased *FGF2* mRNA expression and cluster formation [144]. 

Summarized, inflammatory cytokines play an important role in human OA AC catabolism and inflammation, but it appears that they cannot be responsible for the observed OA AC proliferation, which is a hallmark of early OA. In contrast, OA AC proliferation can only be attributed to a variety of growth factors. Remarkably, of all growth factors discussed in this chapter, only FGF2 is able to concomitantly upregulate proliferation as well as induce catabolic and inflammatory gene expression, which may represent a so far not considered yet potentially important novel concept for onset of inflammation in human OA. 

## 6. MiRNA Regulation of Fibroblast Growth Factor 2, Transforming Growth Factor β and Inflammatory Cytokine Signaling Pathways

During the last years a myriad of publications addressing the differential regulation and effects of miRNA in human OA AC compared to normal AC have been published, which opened new insights into the intensive regulation and crosstalk of signaling pathways. This chapter focuses on those miRNAs with demonstrated regulatory effects in human OA AC, compared to normal AC, and with established targets in FGF2, inflammatory cytokine or TGF-β signaling pathways (see Figure 2).

Concerning miR-9, most studies report an enhanced abundance in OA AC. Both miR-9 and *IL-6* have been reported to be upregulated in damaged hip OA AC compared to undamaged AC areas of the same patients. Furthermore, rIL-1β and rIL-6 may induce miR-9 expression in monolayer cultures of hip OA AC cells. In the same study, *monocyte chemoattractant protein-induced protein 1* (*MCPIP-1*), a post-transcriptional repressor of IL-6 mRNA, has been established as miR-9 target [145]. In a second study, moderate upregulation of miR-9 in hip OA AC compared to age matched healthy AC has been reported [146]. Also, another group documented upregulation of miR-9 expression in both AC and bone of knee OA patients compared to healthy cartilage and bone from donors of the same age. Interestingly, miR-9 reduces basal and also rIL-1β induced MMP-13 protein expression in primary AC cells [147]. Yet, downregulation of miR-9 expression in human knee OA AC compared to age matched normal AC has been shown [148]. In this study, *NF-κB1* has been established as a direct target of miR-9. In liver fibrosis, TGF-β1 downregulates miR-9 expression by promotor methylation, whereas *TGFBR1* (ALK5) and *TGFBR2* have been established as direct targets of miR-9 [149]. Therefore, miR-9 seems to fine-tune inflammatory cytokine signaling, whereas anabolic TGF-β signaling is attenuated.

MiR-16 levels are upregulated in the plasma of knee OA patients [150]. Also knee and hip AC of OA patients exhibit increased miR-16 expression compared to healthy AC [151,152]. *SMAD3* has been determined as a direct target of miR-16, implicating this miRNA in the switch to catabolic TGF-β signaling [152]. Notably, *FGF2* is a direct transcriptional target of miR-16 in human nasopharyngeal carcinoma cells [153], indicating additional repression of FGF2 signaling by this miRNA.

MiR-21 expression is elevated in human OA AC. Indeed, miR-21 suppresses chondrogenesis by directly targeting *growth differentiation factor 5* (*GDF5*), whereas NF-κB signaling is induced [154]. Another direct target of miR-21 is *tissue inhibitor of metalloproteinases 3* (*TIMP3*). In HUVEC, miR-21 dependent downregulation of *TIMP3* coincided with increased MMP-2 and MMP-9 mRNA, and protein expression [155]. Interestingly, rIL-6-induced signal transducer and activator of transcription 3 (STAT3) activation has been implicated in increased miR-21 and miR-181 expression and malignant transformation of a human mammary epithelial cell line by constitutively activating NF-κB signaling [156]. Moreover, miR-21 has been implicated in several chronic diseases related to an aging-dependent increase of inflammation [157]. Collectively, this indicates a contribution of miR-21 to catabolic NF-κB signaling and MMP activation in response to inflammatory cytokines.

MiR-23a is another miRNA directly suppressing *SMAD3* expression, alleviating anabolic TGF-β signaling. Hypomethylation of the promoter region of miR-23a may contribute to its increased expression, which is observed for both, miR-23a and miR-23b in human hip and knee OA AC compared to healthy AC [151,158]. Yet, another study shows downregulation of miR-23a expression in human knee OA AC explant cultures upon rIL-1β treatment, whereas miR-23 expression and release were enhanced in synovial explants from OA patients [159]. This indicates an opposing scenario in rIL-1β treated ex vivo OA AC cultures compared to the in vivo observed upregulation.

Decreased abundance of miR-26a and miR-26b has been detected in human knee and hip OA AC compared to normal AC [151,160,161]. Notably, increasing body mass index (BMI) and NF-κB pathway activity in OA patients has been related to progressive miR-26a downregulation [162]. Direct targets for miR-26a and miR-26b dependent suppression are *karyopherin subunit alpha 3* (*KPNA3*) that modulates NF-κB p65 translocation [161], *high mobility group protein A1* (*HMGA1*), and *mucosa-associated lymphoid tissue lymphoma translocation protein 1* (*MALT1*), which are also involved in positive regulation of NF-κB signaling and *IL-6* expression [163]. Moreover, *TAK1* and *TGF-β activated kinase 1 and MAP3K7 binding protein 3* (*TAB3*), two additional positive regulators of NF-κB signaling are directly targeted by miR-26b [164]. Besides, *SMAD1* and *SMAD4* are directly repressed by miR-26a [165]. Also, *CCN2* is a target of miR-26a [166]. Therefore, miR-26 family downregulation activates NF-κB signaling, promotes catabolic TGF-β signaling, and probably also interferes with FGF2 signaling.

MiR-27b, which directly targets *MMP-13*, is downregulated in human knee OA AC, compared to AC from young and healthy AC donors [167]. Hydrostatic pressure increases both miR-27a and miR-27b expression specifically in human hip OA AC monolayer cell cultures, but not in cell cultures derived from normal hip AC [168]. Remarkably, though rIL-1β downregulates miR-27a and miR-27b in human late stage knee OA AC explant cultures, in synovial explant cultures of patients with late stage knee OA AC an upregulation and enhanced secretion of miR-27a and miR-27b was detectable upon rIL-1β stimulation [159]. Long non-coding RNA-cartilage injury-related (lncRNA-CIR), which is upregulated in OA AC, acts as a sponge for miR-27, whereas miR-27 directly represses lncRNA-CIR expression [167]. In addition, in human chondrosarcoma cells, expression of miR-27b is negatively regulated by adiponectin (ADPN), an adipokine [169]. This indicates a differential expression of miR-27 family members in OA AC and synovium in response to rIL-1β, whereas enhanced catabolic *MMP-13* expression in may be reinforced by adipokines.

The human miR-29 family consists of three mature members, miR-29a, miR-29b, and miR-29c. Although, their targets are largely overlapping, differential regulation has been reported [170]. Indeed, upregulation of all three miR-29 members in human hip OA AC compared to normal AC has been reported, whereas serial passaging of OA AC cells in monolayer culture leads to miR-29 downregulation [171]. In addition, miR-29c is upregulated in the plasma of human knee OA patients compared to healthy controls [150]. Yet, another study documents miR-29a downregulation in human hip and knee OA AC compared to healthy AC and also shows a negative correlation of miR-29a expression with increasing BMI, whereas IL-1β abundance positively correlates with increasing BMI [151]. Interestingly, rFGF2 can increase miR-29a and miR-29b expression in first passage monolayer cultures of human knee AC [172], whereas both, NF-κB and SMAD3 have been implicated in repression of miR-29 family members [170]. rTGF-β1 reduces miR-29 level in human primary OA AC cell cultures. Yet, while NF-κB inhibits miR-29 expression, rIL-1β increases miR-29a and miR-29b expression in a p38 MAPK dependent manner [171]. Indeed, rTNF-α has been identified as miR-29b suppressor in the human chondrosarcoma cell line SW1353 in another study [173]. Notably, miR-29c directly suppresses *MMP-2* expression in human lung cancer cells [174]. Moreover, several collagen genes are predicted targets of the miR-29 family [175] and especially *COL2A1* is repressed by miR-29b, whereas *COL10A1* is lacking a binding site [176]. Additionally, the miR-29 family has been implicated in alterations of DNA methylation and stem cell exhaustion, which is observed during aging [157]. The fact that miR-29 is upregulated in human OA AC suggests a greater effect of FGF2 and inflammatory cytokine regulated MAPK signaling on miR-29 regulation than of NF-κB, as one would expect a miR-29 upregulation under predominating FGF2 and inflammatory cytokine regulated MAPK signaling and a miR-29 downregulation under predominating NF-κB activation. Especially *SMAD3* is targeted by other upregulated miRNAs in human OA AC, suspending negative regulation by TGF-β signaling. Therefore, miR-29 family members may actively contribute to catabolic ECM remodeling by promoting a collagen II/X imbalance in OA AC.

MiR-30a is upregulated in primary AC cells from knee OA patients compared to young healthy donors [177]. Also, a second family member, miR-30b is overexpressed in human hip and knee OA AC compared to healthy AC [151,178]. Moreover, miR-30b abundance is elevated in the plasma of human knee OA patients compared to healthy controls [150]. *SRY-related HMG box-containing 9* (*SOX9*) is a direct target of miR-30a [177], whereas miR-30b targets the *ETS-related gene* (*ERG*) [178], both reducing anabolic mRNA expression. Yet, also *ADAMTS-5* is a direct target of miR-30a [179]. Notably, rIL-1β represses miR-30a expression in monolayer cell cultures established from normal AC and OA AC by recruiting of AP-1 to the miR-30a promoter [179]. Therefore, in human OA AC the miR-30 family is apparently involved in inhibition of anabolic matrix deposition, whereas it is not pro-catabolic. However, the mechanism of miR-30a upregulation in OA AC remains elusive. 

Both miR-33a and its host gene *sterol regulatory element-binding protein 2* (*SREBP-2*) are upregulated primary cell cultures of human knee OA AC compared to healthy AC. Treatment of monolayer cultures of human OA AC cells with rTGF-β1 increased expression miR-33a. MiR-33a reduced *ATP-binding cassette transporter A1* (*ABCA1*) and *apolipoprotein A1* (*APOA1*) mRNA expression levels, which are both involved in cholesterol efflux and elevated *MMP-13* expression levels. While *ABCA1* contains a miR-33 target sequence, the other effects are rather indirect. Indeed, in OA AC reverse cholesterol transport appears to be reduced [180].

Both miR-34a and miR-34b are upregulated in human knee OA AC compared to normal AC [147,181]. Also primary cell cultures of human knee OA AC show increased miR-34a expression compared to healthy controls [182]. In AC, miR-34a targets *sirtuin 1* (*SIRT1*), which is involved in epigenetic gene silencing [182]. Another target is *cysteine-rich angiogenic inducer 61* (*CYR61*), which inhibits ADAMTS-4. Indeed, upregulation of miR-34a by rIL-1β promotes ADAMTS-4 expression in human AC cells [181]. In human primary immortalized fibroblasts the MAPK activated transcription factor ELK1 is involved in miR-34a expression [183]. In prostate cancer cell lines, miR-34b significantly inhibits protein expression of TGF-β, TGF-βR1 and p-SMAD3, but does not affect mRNA level indicating translational repression [184]. Additionally, miR-34 family members have been implicated in altered DNA damage response and telomere shortening associated with cellular senescence [157]. Therefore, this miRNA family promotes catabolic gene expression and contributes to global repression of TGF-β signaling with a focus on the anabolic SMAD3 pathway.

MiR-105 is downregulated by rFGF2 in cell cultures established from human healthy knee AC. Mechanistically, the p65 subunit of NF-kB is implicated in FGF2-mediated miR-105 downregulation. *RUNX2*, involved in the transcription of *ADAMTS-4*, *ADAMTS-5*, *ADAMTS-7* and *ADAMTS-12*, has been identified as direct target of miR-105 [172]. Moreover, *SOX9* is a direct target of miR-105 in human glioma cells [185]. This indicates that miR-105 acts both anti-anabolically and anti-catabolically, whereas its compensatory effect is alleviated by its downregulation in OA.

MiR-125b, which targets *ADAMTS-4*, is downregulated by rFGF2 [172,186]. In human OA AC miR-125b is repressed compared to healthy AC. In addition, an age dependent decrease of miR-125b abundance in human AC has been observed [186]. This indicates an anti-catabolic role of miR-125b in AC, which is attenuated by its downregulation during aging and onset of OA.

MiR-126 has been reported to be upregulated in the plasma of human knee OA patients compared to healthy controls [150]. Yet, downregulation of miR-126 in healthy and OA AC samples from old patients, compared to AC from young, healthy patients has been detected in another study [187]. In HUVEC cells, the MAPK activated transcription factor avian erythroblastosis virus E26 oncogene homolog 1 (ETS1) has been implicated in miR-126 expression [188]. Though, in human glioma cells, *Kirsten rat sarcoma viral oncogene homolog* (*KRAS*) has been identified as direct miR-126 target, indicating negative regulation of the ERK pathway [189]. Therefore, downregulation of miR-126 may reinforce MAPK signaling, while its upregulation may prevent over-activation depending on the context.

MiR-127 expression is reduced in knee OA AC compared to normal AC [190,191]. Increased *MMP-13* expression upon rIL-1β treatment in monolayer cell cultures of human OA AC correlated with miR-127 suppression, with *MMP-13* as a direct target of miR-127. In addition, miR-127 inhibits also *MMP-1* expression in response to rIL-1β [190]. Collectively, with downregulation of miR-127 in OA AC, another anti-catabolic miRNA in AC is disenabled. 

MiR-139 is specifically upregulated in the macroscopically degenerated areas of knee OA AC, compared to macroscopically intact appearing AC from the same patient [192]. rIL-1β and rIL-6 increase miR-139 level OA AC cell cultures, whereas inhibition of miR-139 markedly reduces *IL-6* mRNA and protein expression. *MCPIP1*, a post-transcriptional repressor of *IL-6* mRNA, is a direct target of miR-139. Beyond, *ADAMTS-4* and *MMP-13* mRNA expression is significantly increased by miR-139 mimic. This indicates the involvement of miR-139 in inflammatory cytokine-mediated catabolic gene expression in advanced OA.

Downregulation of miR-140 in human knee and hip OA AC compared to normal AC has been reported [151,161,193]. In cell cultures of knee OA AC and synovial fluid, expression of miR-140 negatively correlates with OA severity [194]. Yet, hydrostatic pressure increases miR-140 expression in human hip OA AC monolayer cell cultures [168]. *MMP-13* [195] and *IL-6* [196] are direct targets of miR-140. In the human rib cartilage cell line C28/I2 rIL-1β reduces miR-140 expression [195]. In contrast, TGF-β signaling can induce miR-140 expression via SMAD3, whereas *SMAD3* is also a target of miR-140 [197]. Summarized, the anti-catabolic miR-140 is repressed in human OA AC and this repression may be mediated by inflammatory cytokines, whereas the TGF-β-dependent miR-140 expression is alleviated by SMAD3 depletion.

MiR-145, upregulated in aged knee OA AC compared to normal AC from younger donors, can be induced by rIL-1β in monolayer cell cultures established from normal and OA AC, with stronger induction of miR-145 observed in OA AC cells [198]. Yet, others documented the downregulation of miR-145 in late stage OA AC, compared to early stage OA AC of the same patients [199] or normal AC [200]. Indeed, in human OA AC increased TNF-α levels correlated with a reduced miR-145 abundance [199]. *SMAD3* is a direct target of miR-145 [198]. Also, human *SOX9* is directly downregulated by miR-145 in cell cultures from human normal knee AC, while the target sequence is not conserved in murine *Sox9*. Notably, in human AC cell cultures miR-145 expression significantly increases from P0 to P2 concomitantly with dedifferentiation [201]. Another miR-145 target in human AC cell lines is *tumor necrosis factor receptor superfamily member 11b* (*TNFRSF11B*). TNFRSF11B downregulation originates upregulation of Collagen II, V and X and reduction of MMP-1, MMP-8 and MMP-13 proteins [200]. Moreover, *A disintegrin and metalloproteinase domain-containing protein 17* (*ADAM17*) can be directly targeted by miR-145 [202], which initiates a negative feedback loop involving the ADAM17 substrate TNF-α, which is upregulated and subsequently reduces miR-145 expression in renal carcinoma cell lines [203]. In human adipocytes miR-145 increases both glycerol release and TNF-α secretion via activation of NF-κB signaling [202]. Therefore, inflammatory cytokine signaling both positively and negatively interferes with miR-145 abundance, with increasing inflammation probably depleting miR-145 levels. Hence, miR-145 is involved in anti-anabolic and catabolic signaling during OA progression.

In human late stage OA AC cells miR-146a is upregulated, while during chondrogenesis of human bone MSPCs downregulation of miR-146a is observed [204]. Increased expression of miR-146a has been also detected in the plasma of human knee OA patients compared to healthy controls older than 40 years [150]. Moreover, miR-146a expression is elevated in peripheral blood mononuclear cells (PBMC) from late stage OA patients [205]. Mechanical pressure injury increases miR-146a abundance in human healthy AC cells, wherein *SMAD4* has been identified as direct target of miR-146a [206]. In addition, hydrostatic pressure increases miR-146a expression in human hip OA AC monolayer cell cultures, which exhibit reduced basal miR-146a level compared normal hip AC cells [168]. However, transfection of synthetic miR-146a dose-dependently antagonized rIL-1-mediated suppression of both *ACAN* and *COL2A1* expression in cells isolated from human early OA AC. Moreover, rIL-1 induced expression of *MMP-13* and *ADAMTS-5* in human early OA AC cells is significantly suppressed by miR-146a [207]. In THP-1 cells, rIL-1β and rTNF-α induce miR-146a expression mediated by NF-κB. *TNF receptor-associated factor 6* (*TRAF6*) and *IL-1 receptor-associated kinase 1* (*IRAK1*) have been identified as direct miR-146a target genes in these cells [208]. Interestingly, lentiviral overexpression of miR-146a increases FGF2 secretion of HUVECs by upregulation of fibroblast growth factor binding protein 1 (FGFBP1) expression via directly targeting *CAMP responsive element binding protein 3 like 1* (*CREB3L1*) [209]. Moreover, miR-146a has been implicated in several chronic diseases related to aging dependent increase of inflammation [157]. Therefore, miR-146a is apparently fine-tuning inflammatory cytokine, TGF-β and FGF2 signaling to prevent over-activation of inflammatory and catabolic pathways in advanced OA.

MiR-149 is significantly downregulated in human knee OA AC and micropellet cultures from OA AC, compared to normal AC. Yet, in bone it is concurrently upregulated [147,210]. In the chondrosarcoma cell line SW1353 both rIL-1β and rTNF-α reduce miR-149 abundance, whereas *IL-6*, *IL-1β* and *TNF-α* are direct targets of miR-149 [173]. Therefore, inflammatory cytokine mediated downregulation of miR-149 appears to be a self-reinforcing system to promote inflammation in advanced OA.

MiR-181a expression is increased in monolayer cell cultures isolated from aged OA AC compared to cells from aged healthy AC. Yet, specifically in cells derived from OA AC hydrostatic pressure downregulates miR-181 [211]. In human knee AC cell monolayer cultures transfection with miR-181 mimic decreases proliferation and increases apoptosis. Moreover, activity of MMP-2 and MMP-9 is enhanced by miR-181 mimic [212]. In addition, elevated miR-181a expression is associated with successful chondrogenesis of human MSPCs [213]. However, there are also studies showing a decreased expression of miR-181 family members, including miR-181a, in OA AC cell monolayer cultures, compared to healthy AC cells from younger donors [191,214]. Notably, in acute myeloid leukemia (AML) cells *KRAS*, *neuroblastoma RAS viral oncogene homolog* (*NRAS*) and *ERK2* have been identified as direct targets of miR-181a [215]. STAT3, activated by inflammatory cytokine signaling, activates miR-181b in human MCF10A cells [216]. Apparently, the miR-181 family promotes catabolic gene expression, while repressing ERK MAPK signaling.

MiR-186 is upregulated in the plasma of human knee OA patients, compared to healthy controls [150]. In human AC, no direct regulation or transcriptional targets have been identified to date. Remarkably, in human THP-1 macrophages miR-186 enhances secretion of IL-6, IL-1β and TNF-α as well as lipid accumulation via targeting *cystathionine-γ-lyase* (*CSE*) [217]. Moreover, in human glioblastoma cells *FGF2* and the *NF-κB* subunit RelA have been identified as miR-186 targets [218]. Summarized, despite induction of inflammatory cytokine expression by miR-186, the inflammatory response is apparently attenuated via NF-κB depletion.

MiR-210 expression is downregulated in human knee and hip OA AC compared to normal AC [151,219]. Transfection of a miR-210 precursor in human OA AC derived monolayer cell cultures induces *COL2A1* mRNA expression, whereas *COL10A1* and *MMP-13* expression is significantly reduced [219]. In human cervical cancer cells, *SMAD4* has been identified as a direct target of miR-210 [220]. Hypoxia-inducible factor-1α (HIF-1α) can induce miR-210 expression in various human cell lines [221]. Notably, in synovial fibroblasts from OA patients rCCN2 induces VEGF secretion by raising miR-210 expression which involves phosphatidylinositol 3-kinase (PI3K)-AKT, ERK, and NF-κB/ELK1 signaling [222]. This indicates that miR-210-mediated anabolic and anti-catabolic signaling is alleviated in human OA AC, whereas in OA synovium miR-210 acts a pro-angiogenic factor.

In human knee OA AC, miR-221 expression is downregulated with an increasing Mankin score. rIL-1β reduces miR-221 expression in monolayer cultures of human OA AC cells. *Stromal cell derived factor 1* (*SDF1*), also known as *C-X-C Motif Chemokine Ligand 12* (*CXCL12*), has been determined as direct target of miR-221 [223]. Moreover, miR-221 is downregulated in synovial fibroblasts derived from patients with OA of the temporomandibular joint (TMJ), compared to synovial fibroblasts of healthy donors. In addition, treatment with rIL-1β suppresses miR-221 expression in TMJ OA synovial fibroblasts. *ETS1*, a transcription factor involved in *MMP-1*, *MMP-2* and *MMP-9* expression, has been identified as direct miR-221 target in TMJ OA synovial fibroblasts [224]. Interestingly, increasing BMI has also been linked to reduced miR-221 abundance [225]. Therefore, reduction of miR-221 abundance by inflammatory cytokines may reinforce their catabolic target gene expression, which, in addition, appears augmented by obesity.

MiR-365 is upregulated in human late stage knee OA AC, compared to macroscopically intact cartilage from the same patients. In cell cultures established from macroscopically normal OA AC, both cyclic loading and rIL-1β increase miR-365 expression by a mechanism involving NF-κB. Yet, hydrostatic pressure reduces miR-365 expression in human hip OA AC monolayer cell cultures [168]. *Histone deacetylase 4* (*HDAC4*) is a direct target of miR-365 and its downregulation has been implicated in catabolic *MMP-13* and *COL10A1* expression [226]. Interestingly, also *IL-6* is a direct target of miR-365 [227]. Therefore, miR-365 is involved in catabolic gene expression, but its inflammatory cytokine induced overexpression may also alleviate inflammatory gene expression in a feedback loop.

Expression of miR-411 is reduced in human knee OA AC compared with normal AC. rIL-1β represses miR-411 in the human immortalized juvenile costal chondrocyte cell line C28/I2. Moreover, *MMP-13* has been identified as direct target of miR-411 and overexpression of miR-411 mimic increases both COL2A1 and COL4A2 at mRNA and protein level [228]. This indicates an anabolic and anti-catabolic function of miR-411, which is apparently overridden by inflammatory cytokines. 

Upregulation of miR-483 has been observed in human knee and hip OA AC, compared to normal AC [151,229]. Also, OA AC micropellet cultures have higher miR-483 level, compared to normal AC micropellet cultures [210]. TGF-β1 is downregulated by overexpression of miR-483 mimic in monolayer cultures from human normal knee AC, which coincides with *COL2A1* and *ACAN* mRNA depletion and *RUNX2* and *MMP13* upregulation [230]. In human MSPC miR-483 directly targets *SMAD4*, which suppresses chondrogenesis. In mice, *matrilin 3* (*Matn3*) and *Timp2* have been identified as direct miR-483 targets [229]. Summarized, miR-483 negatively regulates TGF-β signaling and apparently acts anti-anabolic and pro-catabolic in OA AC.

In monolayer cell cultures derived from human knee OA AC miR-488 expression is reduced, compared to normal AC cell cultures [231]. Exposure to rIL-1β reduces and rTGF-β3 increases miR-488 abundance in normal AC cell cultures. Thereby, miR-488 inhibits MMP-13 activity through targeting the zinc-ion transporter *Zrt- and Irt-like protein 8* (*ZIP8*) [231]. Interestingly, ZIP8 expression can be induced through the canonical NF-κB pathway, which is activated in response to several inflammatory cytokines. Increased intracellular zinc level activate metal regulatory transcription factor-1 (Mtf-1), which positively regulates Mmp-3, Mmp-9, Mmp-13, and Adamts-5 mRNA and protein expression in murine AC monolayer cell cultures [232]. In short, miR-488 obviously exerts its anti-catabolic function by reduction of intracellular zinc availability. Yet, its inflammatory cytokine-mediated downregulation permits catabolic gene expression and MMP activity.

This chapter summarizes the current evidence concerning 27 miRNAs, respectively miRNA families, with a significant up- or downregulation in human OA AC, compared to normal AC. As illustrated in Figure 2, differentially regulated miRNAs interfere with inflammatory cytokine, FGF2 and TGF-β downstream signaling, which, additionally, exhibit an intense crosstalk at the protein level. In human OA AC expression of pro-catabolic target genes is mediated by NF-κB, MAPK and SMAD signaling, whereas pro-inflammatory targets are activated downstream of MAPK and NF-κB signaling. Though proliferation prevails during early OA, expression of catabolic ECM degrading proteins is concomitantly induced. Advanced stages of OA are characterized by sustained activation of inflammatory cytokine signaling, whereas proliferation ceases. Regarding the here discussed miRNAs, there is primarily a net upregulation of IL-6, whereas both positive and negative regulation of NF-κB signaling is reported. Moreover, in human OA AC upregulation of CCN2 and significantly enhanced activation of ERK, JNK and p38 MAPK pathways has been documented. Yet, evidence for ERK signaling regulation by miRNAs is contradictory. In contrast, TGF-β signaling is globally downregulated as a consequence of miRNA presence, especially with a negative regulation of the anabolic SMAD3-mediated pathway, both on protein and miRNA level. In addition, at miRNA level, particularly a reversal of negative regulation of MMP-13 is present in OA AC, which might be also due to many studies specifically examining MMP-13, since several other MMPs are upregulated at protein level, whereas no miRNA-dependent regulation has been examined to date.

## 7. Conclusions

This review focused on the following question: which of the three key events in early OA AC—proliferation, ECM degradation, and inflammation—are inducible by growth factor signaling, inflammatory cytokine signaling, and/or miRNA regulation. Additionally, we aimed to reveal in which sequence(s) these processes can and cannot occur, and whether we can identify a single factor that is able to induce all of these key processes, according to the currently available knowledge. In this context, it is relevant to summarize that differentiated chondrocytes in the SZ of human normal adult AC are predominantly arranged in string patterns embedded in the PCM [12,13] (see Figure 1). Here, growth factors including TGF-β, BMP, and IGF signaling effects mediate AC maintenance-associated anabolic PCM component deposition. However, a hallmark of early OA is proliferation of cartilage-inherent cells [10,13,14], during which the cellular organization changes from single strings to double strings and eventually to small cell clusters [12,233]. During this proliferative phase, the PCM is progressively degraded, apparently by MMPs and other catabolic factors [13]. The proliferating cells may be either dedifferentiated chondrocytes reentering cell cycle or resident or immigrated MSPCs [12,13,23,233]. Whether the proliferation in OA AC is associated with an attempt of cartilage intrinsic anabolic repair or rather a prerequisite for macroscopic cartilage degradation due to a simultaneously lack of extracellular matrix (ECM) maintenance, respectively proliferation-associated degradation, remains elusive. As discussed, proliferation in early OA AC is obviously dependent on FGF2, TGF-β, WNT and NOTCH signaling [13,63,74,105], whereas catabolic gene expression may be induced by FGF2, a switch in TGF-β signaling, and inflammatory cytokines including IL-6 [30,39,60,134]. Importantly, according to what is known, inflammatory cytokines do not induce but in fact suppress human AC cell proliferation [36,128], which in turn means that the proliferative phase during early OA is probably not inducible by inflammatory cytokines and occurs prior to inflammation, whereas catabolic ECM degradation is already apparent during the proliferative phase but steadily increasing with OA severity. Thus, the specific sequence of OA key events is described best by a very early phase of proliferation of human articular cartilage (AC) cells and concomitant anabolic/catabolic effects that are accompanied by incipient pro-inflammatory effects.

Remarkably, it was highly interesting to ask the question whether it was possible to identify any cytokine or growth factor that is potentially able to induce or reinforce all three key events promoting early OA onset and progression. In detail, in human OA AC, proliferation and anabolism, but also catabolism, are associated with TGF-β effects, depending on the receptor utilization. Proliferation and anti-catabolism are associated with canonical WNT/β-catenin signaling effects, whereas non-canonical WNT signaling may contribute to catabolic gene expression. In normal adult AC, proliferation and anabolism are associated with IGF-1 effects. NOTCH signaling contributes to proliferation in human OA AC, whereas it likely inhibits catabolic and inflammatory gene expression. BMP signaling outcome in human AC is anabolic and via a cross-talk with WNT signaling potentially also catabolic. For Hh signaling there are only very few data for human adult AC available. Yet, a potential catabolic role may be assumed. Catabolism is also associated with VEGF signaling in human OA AC. Neither for BMP nor for VEGF experimental evidence reveals a pro-proliferative effect in human AC. In human AC, both catabolism and reinforced pro-inflammatory effects are associated with inflammatory cytokines; yet, proliferation is demonstrably inhibited. Interestingly, based on currently available data, proliferation, catabolism, and pro-inflammatory effects in human AC are solely associated with FGF2. Thus, many factors are able to induce one or two of these three events examined but, importantly, FGF2 was identified as a unique factor capable of concomitantly inducing all three key events. FGF2 is not only involved in proliferative and catabolic gene expression but also in the mRNA expression of the inflammatory cytokines *TNF-α*, *IL-1β*, *IL-6*, *IL-8,* and *MCP-1* [32]. Thus, FGF2-promoted MAPK and NF-κB signaling appears to be uniquely able to induce self-reinforcing inflammation in human adult AC, which is a hallmark of late OA. Therefore, FGF2 is the only cytokine that we can implicate in both the proliferative aspect seen in early OA and also in the degradative and inflammatory progression of later OA. According to the reviewed literature, these properties appear unique to FGF2, as this role cannot be assumed by any other growth factor or inflammatory cytokine. 

This review focused on growth factor-, inflammatory cytokine-, or differential miRNA expression-induced signaling effects in the context of human *primary* osteoarthritis. However, it is important to mention that AC degeneration due to acute injury or due to long-standing mechanical problems such as anterior cruciate rupture or laxity, meniscal damage, and/or joint malalignment is known to lead to *post-traumatic* osteoarthritis (PTOA). Many differences between OA and PTOA are known; we refer to the PTOA literature [234,235]. The here discussed data have not been derived from studies that had PTOA in mind; e.g., models of mechanical overload such as those described in [236,237], or studied therapeutic options after AC injury [238,239,240]. Thus, it remains unclear whether the here derived insights are valid under conditions known to lead to PTOA and designated studies using standardized injury models are needed.

Another important point is that the here discussed signaling effects should not be viewed as isolated events in AC, as several adjacent tissues, including meniscal fibrocartilage, synovium, fat, and bone, with each tissue having its own genetic propensity for anabolic, catabolic, or pro-inflammatory responses, may affect AC by secreted factors. Nevertheless, we focused in this review solely on human AC to produce a systematic foundation for events occurring in human AC, to which the other tissues; e.g., as cytokine and miRNA molecule sources may also contribute.

Collectively, the here presented view represents a novel molecular concept to interpret early OA signaling. However, designated experimental evidence is needed for its confirmation and for judging its potential value in developing novel therapeutic or preventive avenues. 

## Abbreviations

AAV	*adeno-associated virus*
ABCA1	ATP-binding cassette transporter A1
AC	articular cartilage
ACAN	Aggrecan
ACVRL1	activin A receptor like type 1
ADAM17	A disintegrin and metalloproteinase domain-containing protein 17
ADAMTS	A disintegrin and metalloproteinase with thrombospondin motifs
ADPN	adiponectin
ALK	activin receptor-like kinase
AML	acute myeloid leukemia
AP-1	activator protein 1
APOA1	apolipoprotein A1
ASPN	asporin
BMI	body mass index
BMP	bone morphogenetic protein
CaMK2	calmodulin-dependent protein kinase 2
CCL2	C-C motif chemokine ligand 2
CCDN1	cyclin D1
CDK6	cyclin dependent kinase 6
cFOS	cellular oncogene Fos
CIR	cartilage injury-related
COL2A1	collagen type II α 1 chain
COL4A2	collagen type IV α 2 chain
COL10A1	collagen type X α 1 chain
CREB3L1	CAMP Responsive Element Binding Protein 3 Like 1
CSE	cystathionine-γ-lyase
CTGF	connective tissue growth factor
CYR61	cysteine-rich angiogenic inducer 61
CXCL12	C-X-C Motif Chemokine Ligand 12
DKK1	dickkopf WNT signaling pathway inhibitor 1
DKK3	dickkopf WNT signaling pathway inhibitor 3
DLL1	Delta-like 1
DZ	deep zone
ECM	extracellular matrix
ELF3	E74-like factor 3
ELK1	ETS-domain protein ELK1
ERG	ETS-related gene
ERK	extracellular signal-regulated kinase
ETS	avian erythroblastosis virus E26 oncogene homolog
FGF2	fibroblast growth factor 2
FGFBP1	fibroblast growth factor binding protein 1
FGFR	fibroblast growth factor receptor
FLT-1	Fms related tyrosine kinase 1
FLT-4	Fms related tyrosine kinase 4
FOSB	FBJ murine osteosarcoma viral oncogene homolog B
FRZB	frizzled related protein
GDF5	growth differentiation factor 5
GLI1	glioma-associated oncogene homolog 1
HDAC4	histone deacetylase 4
HES	hairy and enhancer of split
HES1	hairy and enhancer of split 1
HES5	hairy and enhancer of split 5
Hh	Hedgehog
HHIP	hedgehog interacting protein
HIF-1α	hypoxia-inducible factor-1α
HMGA1	high mobility group protein A1
HSPG	heparan sulfate proteoglycan
IGF	insulin like growth factor
IHH	indian hedgehog
IL	interleukin
IL-1β	IL-1 beta
IL-1R	IL-1 receptor
IL-1Ra	IL-1 receptor antagonist
IRAK1	IL-1 receptor-associated kinase 1
LncRNA	long non-coding RNA
I-TAC	interferon-inducible T-cell alpha chemoattractant
JAG1	jagged 1
JAK	janus kinase
JNK	JUN N-terminal kinase
KDR	kinase insert domain receptor
KPNA3	karyopherin subunit alpha 3
KRAS	Kirsten rat sarcoma viral oncogene homolog
MALT1	mucosa-associated lymphoid tissue lymphoma translocation protein 1
MAPK	mitogen activated protein kinase
MAP3K7	mitogen-activated protein kinase kinase kinase 7
MCP-1	monocyte chemotactic protein 1
MCPIP-1	monocyte chemoattractant protein-induced protein 1
MIG	monokine induced by interferon-gamma
miRNA	Micro RNA
MMP	matrix metalloproteinase
MSPC	mesenchymal stem and progenitor cells
Matn3	matrilin 3
Mtf-1	metal regulatory transcription factor-1
MZ	middle zone
NFAT5	CaMK2 nuclear factor of activated T-cells 5, tonicity-responsive
NFATC2	nuclear factor of activated T-cells 2
NF-κB	nuclear factor kappa B
NOTCH	notch homolog
NRAS	neuroblastoma RAS viral oncogene homolog
OA	osteoarthritis
OSM	oncostatin M
PBMC	*peripheral blood mononuclear cells*
PCM	pericellular matrix
PI3K	phosphatidylinositol 3-kinase
PKC δ	protein kinase C δ
PTCH1	patched 1
R	recombinant
RAS	rat sarcoma viral oncogene homolog
RUNX2	runt related transcription factor 2
SCSO	superficial cell spatial organization
SDF1	Stromal cell derived factor 1
SF	synovial fluid
SHH	sonic hedgehog
SIRT1	sirtuin 1
SMAD	SMA- and MAD-related protein
SNP	single nucleotide polymorphism
SOX9	SRY-related HMG box-containing
SREBP-2	sterol regulatory element-binding protein 2
STAT3	signal transducer and activator of transcription 3
SZ	superficial zone
TAB3	TGF-β activated kinase 1 and MAP3K7 binding protein 3
TAK1	TGF-β-activated kinase-1
TGF-β	transforming growth factor β
TGFBR	transforming growth factor β receptor
TIMP	tissue inhibitor of metalloproteinases
TMJ	temporomandibular joint
TNF-α	tumor necrosis factor α
TNFRSF11B	tumor necrosis factor receptor superfamily member 11b
TRAF6	TNF receptor-associated factor 6
VEGF	vascular endothelial growth factor
WNT	wingless-type MMTV integration site family
ZIP8	Zrt- and Irt-like protein 8
